# Northwest Texas Medical Association

**Published:** 1897-06

**Authors:** 


### Society Notes.

### Northwest Texas Medical Association.

The Northwest Texas Medical Association convened in Bowie Tuesday afternoon at 3 o'clock at Knights of Pythias Hall. Dr. C. S. Bobo, the president, occupied the chair. Dr. J. L. Gaston, the secretary, was at his post of duty.

Prayer was offered by Rev. J. M. Small.

The minutes of the previous meeting were read and approved.

On motion of Dr. Riley the vacancies on the Judicial Council were filled. The complexion of the new board reads: Drs. Elliott and Riley, Bowie; Dr. Gray, of Fort Worth, and Dr. Lopp, of Dan. The Council returned and reported favorably on the applications for membership of Dr. T. J. Cook, of Crafton; Dr. H. F. Wilson, of Nocona, and Dr. F. T. Beauchamp, of Newark.

Dr. Riley, on membership cards, asked for further time.

The section on obstretics and gynecology was then taken up. Chairman J. F. Robertson being absent, Dr. R. C. Mitchell was called to the chair.

The section was then temporarily suspended, on motion of Dr. Riley, in order to give Dr. Gray, of Fort Worth, an opportunity to read an interesting volunteer paper on "Sympathetic Ophthalmia." Discussed by Drs. J. F. Elliott, B. C. Mitchell and H. Riley, of Bowie. Discussion closed by Dr. Gray. As this paper was an exceptionally able one, the Association asked leave, and was granted the privilege of retaining it, to be preserved by the Association.

The Association then returned to the section on obstetrics and gynecology.

Dr. B. C. Mitchell presented a paper on "Chloroform in Labor." Discussed by Drs. Riley, Elliott and Gaston, of Bowie; Dr. Wilson, of Nocona and Dr. Bobo, of Boyd.

Adjournment was had till 8:30 p. m. Tuesday.

TUESDAY, 8:30 P. M.

Immediately upon receiving the application of Dr. N. S. Crain, of Spanish Fort, for membership, it was favorably reported by the Judicial Council, and he was unanimously elected.

A letter was received from Dr. T. R. Allen, of Greenwood, regretting his inability to be present at the meeting.

Work resumed on section of obstetrics and gynecology. Discussion on Dr. B. C. Mitchell's paper was taken up, and after remarks by Dr. Tye, of Chickasha, I. T.; the discussion on the paper was closed by Dr. Mitchell himself.

A lengthy paper was then read by Dr. C. E. Burns, of Bowie, on "Puerperal Septicæmia." Discussed by Drs. Elliott and B. C. Mitchell, of Bowie, Dr. Tye, of Chickasha, and Dr. Wilson, of Nocona. Closing talk by Dr. Burns.

On motion of Dr. Gaston the Association adjourned to meet at 9 a. m., Wednesday.

WEDNESDAY, 9 P. M.

The Association was promptly called to order by President Bobo.

Dr. Elliott, at request of Dr. Riley, told a story to point a moral and adorn a tale.

The chair then announced as a nominating committee the following doctors: Riley, Sneed, Strong, T. J. Cook, F. D. Beauchamp, J. L. Lopp, T. H. Clark, Crain, Elliott and Burns.

Dr. Riley moved that the annual dues to the Association be $1 per annum. Carried.

An amendment was proposed to the constitution, that the Association convene annually, instead of semi-annually, as is now the case. Lost.

Section on practice was called, and Dr. T. R. Allen being absent, Dr. Lopp was called to preside in this section.

Dr. M. N. Gardner, of Greenleaf, Kan., was received as a member of this Association.

WEDNESDAY, 1:30 P. M.

Under suspension of the rules Dr. B. C. Mitchell reported a clinical case of a little boy. Discussed by Drs. Wilson, Bobo and Garrison.

The election of officers being the next order of business, the following gentlemen were nominated, elected and installed: President, Dr. H. Riley, of Bowie; Vice-President, Dr. J. F. Roberson, of Duncan, I. T.; Dr. R. H. Mitchell, of Bowie, Secretary; Dr. R. P. Tye, of Chickasha, I. T., Treasurer.

Section on practice was opened by Dr. B. C. Mitchell in a reported case.

A paper was read by Dr. Riley entitled "A Few Prescriptions." Discussed by Drs. Garrison, Bobo and B. C. Mitchell. Dr. Riley concluded the discussion.

A paper was read by Dr. Sneed Strong, of Stoneberg, entitled, "Some attainments Necessary to Success in Surgery." Discussed by Drs. Riley, Wilson, Gardner, Jameson, Tye, Beauchamp and Elliott. Dr. Strong closed the discussion.

A motion prevailed to temporarily pass section on surgery and go into executive session.

The Secretary reported the indebtedness at the close of the meeting to be about $30.

An invitation was extended by the Fort Worth Medical Association to have the Association hold its October meeting in that city. The invitation was unanimously accepted.

The secretary was instructed to write each member that his annual fees would be $1.

Dr. Riley inquired of the profession the manner in which they treated hydrocele. It was generally agreed that the injection of Lugol's solution of iodine was the proper treatment.

WEDNESDAY NIGHT'S PROGRAMME.

A large audience, made up of both ladies and gentlemen, gathered at the opera house to witness an interesting feature of the proceeding attending the seventh semi-annual meeting of the Northwest Texas Medical Association. Dr. Riley, the newly-elected President of the Association, acted as master of ceremonies.

Opening Overture.......................................Anvil Chorus

Messrs. Smith, Harper, Wear, and Misses
Burbee and Densler.

Address of Welcome..............................Judge Xaver Ryan

Piano Duet..................................................... ———

Misses Jennie May Densler, Fairy McCurdy.

Response to address of welcome by the retiring president, Dr. C. S. Bobo, of Boyd.

Quartette........................................................ ———

Messrs. Smith and Weir, Mrs. Smith and Miss Burbee.

Interesting paper read by Dr. J. F. Elliott on "Medical Ethics." A discussion of the paper followed.

Local solo with violin obligato, by Messrs. Weir and Harper and Miss Hattie Burbee.

The opening solo was a grand selection from Verdi's opera of "Il Trovatore," introducing the Anvil Chorus. The instrumentation and personnel were as follows: Violin, T. E. Harper; cornet, W. C. Smith; piano, Misses Hattie Burbee and Jennie May Densler; anvil, T. H. Wear. This overature is unsurpassed, and was handled with the skill of professionals.

The piano duet showed unusual musical ability on the part of the young ladies, Misses Densler and McCurdy.

The vocal numbers were a marked success, and go a great way toward proving that Bowie is not far behind the very best cities in point of musical talent.

Taken all in all, the affair was a signal success, and should work to the advantage of the ones taking part, as no profession can succeed without the proper encouragement.

At the close of the evening's entertainment the visiting physicians repaired to Bob's Hotel, where an excellent supper greeted them.

THURSDAY, 8:30 a. m.

The meeting was called to order promptly, with President Riley in the chair.

The following resolutions were then introduced and adopted:

*Resolved*, That we congratulate Dr. Bobo, our retiring President, for his able address, delivered at the opera house; also Dr. Elliott for his magnificent paper on "Medical Ethics." Thanks are also due Dr. Riley for his manly speech of acceptance of the high office of President of the Association.

*Resolved*, That we extend our thanks to the Knights of Pythias for the free use of their splendid hall.

*Resolved*, That the thanks of this Association be extended to the citizens of Bowie for the hospitable manner in which they have entertained us during the meeting; also to the local physicians for their cordial welcome and their courtesy to us during our stay among them.

*Resolved*, That the thanks of this Association be extended to Mr. T. H. Wear, Mr. and Mrs. W. C. Smith, Prof. T. E. Harper and Miss Hattie Burbee and her pupils, Misses Densler and McCurdy, for the excellent music furnished; also that thanks be extended to Miss Myrtle Brown for the lovely boquet of flowers furnished that ornamented our hall during the session.

*Resolved*, That the thanks of the Association are tendered Drs. Gayton and Riley for the magnificent banquet given the members of the Association.

*Resolved*, That thanks be extended mine host Bob Meyer, for the magnificent repast of "Stuffed goose, raw cabbages," etc., on the side.

*Resolved*, That thanks of the Association be and are hereby extended the *Bowie Cross Timbers* for the able and efficient manner in which it reported our proceedings, and for courtesies extended.

J. W. HARVEY,
B. C. MITCHELE,
Committee.

By resolution all the foregoing resolutions were unanimously adorted.

The janitor's account of $4.50 was allowed and ordered paid.

A motion prevailed that the Secretary pay all accounts approved.

A motion prevailed that President Riley be given time to appoint chairmen for sections.

Dr. Gardner made a speech of thanks to the Association for the kind reception extended him. This was followed by a motion that especial mention be made of the doctor's speech at the opera house.

A motion prevailed that the physicians of Fort Worth be notified that the Northwest Texas Medical Association accepts their kind invitation to meet in their city on the first Tuesday in October next.

The meeting was closed with prayer by Dr. B. C. Mitchell.

H. Riley, M. D., President.
R. H. Mitchell, M. D., Secretary.

### ROLL OF MEMBERSHIP.

The following physicians compose it: W. L. York, Decatur, Texas; Bacon Saunders, Fort Worth, Texas; H. Riley, Bowie, Texas; J. T. Sparkman, Alvord, Texas; Wm. C. Dunaway, Little Rock, Ark.; C. C. Davis, Opelika, Ala.; T. R. Allen, Greenwood; B. F. Garrison, Montague; C. W. Parker, Alvord, F. H. Clark, Stoneberg; J. F. Elliott, Bowie; W. D. Patton, Bowie, Texas; J. M. Stephens, Dan, Texas; J. F. Bertram, Chico; J. L. Gaston, Bowie; J. D. Burch, Aurora; R. H. Mitchell, Bowie; B. C. Mitchell, Bowie; O. J. Kendall, Wichita Falls; W. B. Kyle, Keeter, Texas; W. B. Palmer, Audubon, Texas; H. O. Stacy, Quanah; J. V. Clark, Salona, J. R. Floyd, Paradise; E. A. Johnson, Henrietta; J. C. McNeese, Ardmore, I. T.; H. F. Schoolfield, Sunset; W. A. Adams, Fort Worth; F. D. Thompson, Fort Worth; J. M. Gose, Alvord; J. S. Ross; Bowie; J. P. Clark, Stoneberg; Sneed Strong, Stoneberg; Frank Gray, Fort Worth; J. Younger, Post Oak; C. S. Bobo, Boyd; J. Lopp, Dan; R. L. Miller, Decatur; Jno. T. Robinson, Jacksboro; R. J. Wilson, Bellevue; J. E. Stinson, Montague; R. H. Roark, Aurora; P. C. Funk, Bridgeport; W. A. Morton, Paradise; R. P. Tye, Chickasha, I. T.; W. J. Rogers, Belcher; J. E. Dodson, Vernon; D. D. Clark, Montague; J. F. Robertson, Duncan, I. T.; C. E. Burns, Bowie; C. H. Knox, Greenwood;

J. W. Harvey, Sunset; Q. Lipscomb, Denton; J. W. Duncan, Tage; Dr. Long, Duncan, I. T.

NEW MEMBERS.

M. N. Gardner, Greenleaf, Kansas; H. T. Wilson, Nocona; N. W. Crain, Spanish Fort; F. D. Beauchamp, Newark; T. J. Cook, Crafton.

